# Brain Age in Adult Patients With Early‐Treated Phenylketonuria

**DOI:** 10.1002/jimd.70158

**Published:** 2026-02-17

**Authors:** Laura Winiger, Raphaela Muri, Tobias Kaufmann, Michel Hochuli, Emma Vardy, Piotr Radojewski, Katarzyna Pospieszny, Roman Trepp, Regula Everts

**Affiliations:** ^1^ Department of Diabetes Endocrinology, Nutritional Medicine and Metabolism, Inselspital, Bern University Hospital and University of Bern Bern Switzerland; ^2^ Graduate School for Health Sciences, University of Bern Bern Switzerland; ^3^ Translational Imaging Center (TIC), Swiss Institute for Translational and Entrepreneurial Medicine Bern Switzerland; ^4^ Department of Psychiatry and Psychotherapy Tübingen Center for Mental Health, University of Tübingen Tübingen Germany; ^5^ Centre for Precision Psychiatry, Division of Mental Health and Addiction Institute of Clinical Medicine, University of Oslo Oslo Norway; ^6^ German Center for Mental Health (DZPG), partner Site Tübingen Tübingen Germany; ^7^ Department of Geriatric Medicine Royal Oldham Hospital, Northern Care Alliance NHS Foundation Trust Oldham UK; ^8^ Manchester Academic Health Sciences Centre, School of Health Sciences, Faculty of Biology, Medicine and Health, The University of Manchester Manchester UK; ^9^ Support Center for Advanced Neuroimaging (SCAN), University Institute of Diagnostic and Interventional Neuroradiology, Inselspital, Bern University Hospital and University of Bern Bern Switzerland; ^10^ Division of Neuropaediatrics, Development and Rehabilitation, Department of Paediatrics Inselspital, Bern University Hospital and University of Bern Bern Switzerland

**Keywords:** brain age gap, cognition, metabolism, mood, phenylketonuria, PKU

## Abstract

Structural brain alterations have been observed in individuals with phenylketonuria (PKU); however, the potential impact of PKU on brain aging remains unexplored. This study investigated brain age in adults with early‐treated classical PKU compared to healthy controls. Thirty early‐treated adults with classical PKU (age 19–48 years) and 59 age‐, sex‐, and education‐comparable healthy controls underwent structural magnetic resonance imaging (MRI), cognitive and mood assessment, and blood sampling. Brain age was estimated using machine learning models trained to predict brain age from MRI‐derived features across the full brain, cortical lobes, or subcortical regions. The brain age gap (BAG)—the difference between brain age and chronological age—was calculated. In addition, white matter lesion load was rated in patients. While patients with PKU showed differences in BAG for four out of eight brain age estimates, only the BAG in the insula was significantly higher in PKU than controls after correcting for multiple comparisons (*p*_*uncor*_ = 0.006, *η* ^2^ = 0.07). The cingulate BAG was positively associated with concurrent and historical Phe levels (*r*_*s*_ = 0.41–0.69, *p*_*uncor*_ < 0.05) and with white matter lesion load (*r*_*s*_ = 0.40, *p*_*uncor*_ = 0.034). Further, subcortical and cingulate BAG were linked to cognitive performance (*r*_*s*_ = −0.41–0.38, *p*_*uncor*_ < 0.05). These correlations did not survive FDR‐correction. In conclusion, the elevated insular BAG in adults with early‐treated PKU may reflect atypical brain development due to cumulative effects of early‐life or lifelong metabolic disturbances. Longitudinal studies are warranted to elucidate brain aging trajectories and their cognitive implications in PKU.

## 1 Introduction

Phenylketonuria (PKU) occurs due to a deficiency in the enzyme phenylalanine hydroxylase (PAH) activity, leading to elevated phenylalanine (Phe) concentrations in the blood and neurotoxic levels in the brain [1]. This metabolic disturbance affects both brain structure and function. Previous research has reported reduced whole‐brain volumes, decreased cortical thickness, and more white matter (WM) lesions in adult patients with PKU compared to healthy controls [2, 3, 4]. Structural alterations, such as increased putamen volume, have been positively correlated with blood Phe levels [2]. In contrast, other studies have found no associations, such as between cortical thickness and Phe levels [4]. Alterations in white and gray matter may also be linked to cognitive impairments [5]. However, the relationship between concurrent elevated Phe levels and cognitive outcomes in adulthood remains debated [3, 4, 6], and the long‐term consequences of elevated Phe in adulthood are still unclear [7].

Since the advent of neonatal screening in the 1970s, the early‐treated PKU population is progressively reaching middle and late adulthood, entering their fifth and sixth decades of life. This demographic shift presents a unique opportunity to study the long‐term effects of lifelong metabolic control on brain structure, function, and aging. The potential impact of PKU on brain aging remains poorly understood [8]. Advancements in MRI techniques have greatly enhanced our understanding of healthy brain aging, the age‐related accumulation of changes in the brain, which is typically characterized by region‐specific atrophy, reductions in brain volume and cortical thickness, and cognitive decline [9, 10, 11, 12]. Deviations from normative brain aging have been associated with more pronounced brain atrophy, advanced neural loss, and greater cognitive decline, leading to greater impairments in daily life [9, 13]. Despite a recent comprehensive definition on white matter brain changes over the lifespan [14], questions around factors contributing to suboptimal brain aging remain [15], and to date there is still no clear consensus definition of aging—particularly of biological aging [16, 17].

Brain age can be estimated through the brain age gap (BAG), defined as the difference between predicted brain age and chronological age [18]. Supervised machine‐learning (ML) techniques are widely used to predict brain age from MRI data [19]. Typically, a regression‐based ML model is trained and tested on a large sample of healthy individuals scanned across multiple sites to capture normative age‐related neuroanatomical changes [20, 21]. This model is then applied to new participants to estimate their brain age at the whole‐brain or regional level, such as cortical lobes [20]. ML approaches have demonstrated high accuracy in predicting brain structure from MRI data [21].

Larger BAGs, both globally and regionally, have been associated with poorer physiological and psychological health outcomes [20]. Higher BAG has been reported in neurological (e.g., Alzheimer's disease), psychiatric (e.g., depression, borderline personality disorder), and metabolic conditions (e.g., diabetes mellitus type 2) [10, 20, 22, 23, 24, 25, 26]. Previous studies suggest that accelerated brain aging, reflected in higher BAG, may be linked with poorer cognitive performance and an increased risk of cognitive decline [27, 28]. The BAG serves as a non‐invasive, objective, and quantitative biomarker of brain age and as a tool to assist in diagnostic evaluation and therapeutic decision‐making. The BAG is able to capture meaningful variance in brain structure, despite the multifactorial biological contributors to a predicted brain age which might be influenced by genetics, lifestyle and disease [21]. Nevertheless, it remains unkown, whether deviations from the normative brain aging curve reflect accelerated aging or chronic disease‐specific alterations.

The primary aim of this cross‐sectional study was to investigate the BAG in adult patients with early‐treated classical PKU compared to healthy controls. Furthermore, we explored associations between BAG and cognitive performance, mood, metabolic parameters (e.g., blood Phe levels), and white matter (WM) lesion load. Based on our previous findings [4, 29], we hypothesize that patients with PKU show higher global and regional brain age as a disease‐specific marker. This study aims to provide first insights into brain age and its variability in adult patients with early‐treated PKU, and to deepen our understanding of the relationships between brain age and cognitive functioning, mood, and metabolic control.

## 2 Methods

### 2.1 Participants

Thirty early‐treated adults with classical early‐treated PKU and 59 age‐, sex‐, and education‐comparable healthy controls were recruited for the baseline measurements between July 2019 and May 2022 as part of the PICO study (Phenylalanine and its Impact on Cognition; [30]). The PICO study is a randomized, placebo‐controlled, crossover, noninferiority trial, approved by the Cantonal Ethics Committee Bern, Switzerland (2018‐01609), registered on clinicaltrials.gov (NCT03788343), and adhered to the ethical principles outlined in the Declaration of Helsinki. All participants provided written informed consent before participating in the study.

The patient and control sample included in this study encompassed all participants included in a prior study investigating cross‐sectional structural imaging data [4]. The recruitment of the 30 patients with PKU (13 females, median age = 35.5 years, IQR = 12.3, age range = 19–48 years) was performed by their respective metabolic specialists at metabolic centers across Switzerland, Germany, and Austria. The inclusion criteria for study participation in the PICO study comprised age ≥ 18 years, a positive newborn screening test for PKU, and treatment with a Phe‐restricted diet within the first 30 days of life. Patients were excluded if Phe concentrations exceeded 1600 μmol/L in the past 6 months, they had disrupted their diet in the past 6 months, they had an untreated vitamin B12 deficiency, or if they were currently pregnant or breastfeeding.

The 59 healthy controls (26 females, median age = 30.0 years, IQR = 11.0, age range = 18–53 years), comparable to patients in terms of age, sex, and educational level, were recruited in the environment of Bern and Zurich through advertisements and word of mouth. Exclusion criteria for patients with PKU and controls included severe psychiatric conditions (e.g., schizophrenia), neurological disorders (e.g., multiple sclerosis), inability to follow the study procedures (e.g., lack of fluency in German or French) and conditions interfering with cognitive testing or MRI (for more details see Trepp et al. [6]).

### 2.2 Neuroimaging

Brain MRI was performed following an 8–12 h overnight fast using a 3T Siemens Prisma MRI system equipped with a 64‐channel head coil. The head coil was fitted with an integrated mirror system, enabling participants to view a calming nature documentary during the structural scan acquisition, which was aimed at minimizing motion. Imaging data from all participants were collected at the Translational Imaging Center of the Swiss Institute for Translational and Entrepreneurial Medicine in Bern, Switzerland.

A high‐resolution (1 mm^3^) T1‐weighted image (MPRAGE) was collected for all participants as a basis for the BAG analyses using the following parameters: repetition time TR = 1950 ms, echo time TE = 2.26 ms, inversion time TI = 900 ms, acquisition time TA = 4:34 min, flip angle = 9, in‐plane resolution = 1 × 1 mm, slice thickness = 1 mm, number of slices = 176, field of view = 256 mm × 256 mm, matrix = 256 × 256. Further, T2‐weighted images were acquired (axial: 0.5 × 0.5 × 3.0 mm, TR = 4800 ms, TE = 88 ms, TA = 1:04 min; sagittal: 1.0 × 1.0 × 4.0 mm, TR = 3000 ms, TE = 84 ms, TA = 0:26 min; and coronal: 1.0 × 1.0 × 4.0 mm, TR = 3000 ms, TE = 84 ms, TA = 0:23 min).

### 2.3 Brain Age Gap

Brain age estimation was performed based on a recent implementation [20] using both global and regional MRI‐derived features as inputs. The regional brain age estimations were derived from Freesurfer‐based [31] segmentation of six cortical lobes (cingulate, frontal, insular, occipital, parietal, temporal), as well as a subcortical/cerebellar features set, yielding seven regional models. The global brain age estimation was derived from the combination of all regional sets. The models employed were previously established and validated elsewhere (for details see [20]). In short, they were built using the xgboost package [32, 33] in R, trained on a large cohort and validated using fivefold cross‐validation and tested in various independent samples. Here, we used these pretrained models to estimate eight brain age values for each participant in the PICO study (one global, seven regional), and subsequently calculated the respective BAGs—defined as the difference between estimated and chronological age.

For the purpose of quality controls, relationships between the BAG and chronological age were examined by calculating Spearman rank correlations, and potential sex differences in the BAG were assessed using robust linear regression analyses with rank‐based estimations. Negative uncorrected associations between chronological age and the BAGs occurred in both patients with PKU and controls in the cingulate, the occipital cortex, and subcortical regions. In controls, the insula was additionally negatively associated with chronological age (Table S1a for detailed statistics). Uncorrected sex differences were found in controls, with women showing higher BAGs in the full brain, the insula, and the parietal cortex (Table S1b for detailed statistics). Therefore, we included age and sex as covariates in all further analyses.

Additionally, the reliability of the BAG was evaluated by calculating intraclass correlations (ICCs) of the BAG over three time points across the MRI measurements performed in the framework of the previous randomized interventional trial of the PICO study. These three time points included the first three non‐interventional (Phe‐free) measurements for patients who were assigned to the placebo‐Phe group and only received Phe between time points three and four. Interventional parts were excluded to prevent potential influences of Phe on the measurement of reliability (for more details regarding the study design of the PICO study, see [30]).

Analyses of reliability revealed very good (ICC 0.75–0.90) to excellent (ICC > 0.90) intra‐class correlations across the BAGs in all regions of interest (Table 1).

**TABLE 1:** Intra‐class correlations for the BAG across all brain regions.

Brain region	ICC	95% CI	*p*_*uncor*_
Full brain	0.95	[0.87, 0.98]	< 0.001*
Frontal	0.90	[0.77, 0.97]	< 0.001*
Temporal	0.95	[0.89, 0.98]	< 0.001*
Parietal	0.91	[0.80, 0.97]	< 0.001*
Occipital	0.95	[0.87, 0.98]	< 0.001*
Insula	0.86	[0.70, 0.95]	< 0.001*
Cingulate	0.92	[0.82, 0.97]	< 0.001*
Subcortical	0.94	[0.86, 0.98]	< 0.001*

*Note:* Intraclass correlation coefficients (ICC) for the predicted age in *n* = 13 patients with PKU across the first three Phe‐free time points (T1–T3). The model used is a two‐way model with consistency type. Uncorrected *p*‐values are indicated as *p*_*uncor*_. All correlations in this table remained significant after FDR‐correction. Significant correlations (*p* < 0.05) are highlighted in bold and with an asterisk.

Abbreviations: CI = confidence interval; ICC = Intraclass Correlation Coefficient.

### 2.4 Concurrent Metabolic Parameters

To determine plasma Phe, Tyrosine (Tyr), and Tryptophan (Trp), blood sampling was performed after an 8–12 h overnight fast before the MRI examination in patients with PKU. High‐performance ion‐exchange liquid chromatography with post‐column photometric detection of ninhydrin‐derivatized amino acids was employed.

### 2.5 Historical Metabolic Parameters

Historical metabolic control was assessed using the Index of Dietary Control (IDC), defined as the mean of yearly medians of all available Phe levels throughout a patient's lifetime. Four age categories were established to account for developmental differences: childhood 0–5 years, childhood 6–12 years, adolescence 13–17 years, and adulthood ≥ 18 years. A minimum of 10 Phe measurements per age category was required for IDC calculation; otherwise, the respective category was excluded from analysis [34]. Lifetime Phe concentration was calculated as an additional category. For further details and descriptive statistics on these parameters, see Muri et al. [4].

### 2.6 White Matter Lesions

Two board‐certified neuroradiologists independently assessed WM lesions on anonymized, randomized T_2_‐weighted MRI scans of patients with PKU. Raters were blinded to the patient's medical history, age, and sex. WM lesions were defined as regions of high signal intensity on T_2_‐weighted images and interpreted as a reflection of intramyelinic edema in early‐treated patients [35]. Lesions were rated on a scale from 0 to 12, and a 3‐point scale (0 = no WM changes; 1 = deep WM involvement; 2 = subcortical WM involvement) was used to rate each of four lobes (frontal, parietal, temporal, and occipital). Additionally, the brainstem and cerebellum were each rated on a 2‐point scale (0 = no involvement; 2 = presence of WM abnormalities), in accordance with previously established criteria [36]. A total WM lesion score was calculated for every participant.

### 2.7 Cognitive Assessment

Assessment of cognitive performance was conducted using the test battery of the PICO study [30]. To assess general intelligence, the subtests Matrix Reasoning, Vocabulary, Symbol Search, and Arithmetic from the Wechsler Adult Intelligence Scale, Fourth Edition (WAIS‐IV; [37]) were used. Components of executive functions were measured following the model of Miyake et al. [38]. Executive functions included working memory (accuracy in percentages, measured with the n‐back task of the Test of Attentional Performance (TAP); [39] and number of correct sequences in the respective subtest letter‐number sequencing of the WAIS‐IV), inhibition, and cognitive flexibility (seconds to complete, measured with the Color‐Word Interference Test conditions 3 and 4, respectively; Delis–Kaplan Executive Function System [DKEFS]; [40]), design fluency (total correct designs measured with the design fluency test conditions 1–3; DKEFS), and verbal fluency (total number of correct generated words measured with the verbal fluency test condition letter‐fluency; D‐KEFS). Attention was measured using the TAP subtests alertness (median reaction time in milliseconds), sustained attention (standard deviation of reaction time in milliseconds), and divided attention (total number of omissions; Zimmermann and Fimm [39]). Additionally, manual dexterity was assessed using the respective subtest of the Purdue Pegboard (total number of correctly placed parts in the assembly subtest; [41]).

### 2.8 Mood

The German short form of the Profile of Mood States (POMS; [42]) is a self‐report questionnaire comprising 35 items, in which participants rate the occurrence of various mood states on a 7‐point Likert scale (0 = not at all to 6 = very strongly). The short form includes four subscales: Anxiety (e.g., feelings of inferiority, despair, or anxiety; score range: 0–84), Fatigue (e.g., feelings of exhaustion, listlessness, or lethargy; score range: 0–42), Vigor (e.g., feelings of energy, liveliness, or vitality; score range: 0–42), and Anger (e.g., feelings of anger, irritation, or distress; score range: 0–42). A Total Mood Disturbance (TMD) score can be derived by subtracting the Vigor subscale score from the sum of the other three subscales, with a possible range of −42 to 168 [43, 44]. The POMS demonstrated good to excellent reliability (Cronbach's α = 0.89–0.95; [42]) and an acceptable test–retest reliability for each subscale's scores after seven and after 30 days (0.52–0.80 and 0.32–0.73, respectively; [44]).

Depression was assessed using the revised Beck Depression Inventory (BDI‐II; [45]), a widely used 21‐item self‐report questionnaire. The BDI‐II employs a 4‐point Likert scale (0–3), with total scores ranging from 0 to 63. The total score is the sum of all item scores. In a non‐clinical sample, the German version of the BDI‐II demonstrated good to excellent internal consistency (Cronbach's *α* = 0.89; [46]) and satisfactory test–retest reliability (rtt = 0.78 after 3 weeks and 5 months, respectively; [47]).

### 2.9 Statistical Analysis

All analyses were performed in R version 4.2.2. An analysis of covariance (ANCOVA) was calculated to evaluate possible differences in the BAG between patients with PKU and controls. BAG served as the dependent variable, group as the independent variable, and age and sex were included as covariates. Adjusted means with 95% confidence intervals are reported for each group. All subsequent analyses were non‐parametric due to a lack of normal distribution for cognition, mood, and metabolic parameters.

Relationships between the BAG and cognitive performance, mood, metabolic parameters, and WM lesions were analyzed by calculating partial Spearman rank correlations, accounting for age and sex. Bootstrapping (*n* = 5000) was used to calculate confidence intervals for Spearman correlations. All analyses were performed on a full brain level and across seven brain regions. Brain regions included frontal, temporal, parietal, occipital, insula, cingulate, and subcortical areas. Only results with *p*‐values surviving correction for false discovery rate (FDR; Benjamini and Hochberg [48]) were interpreted as statistically significant. Given the relatively small sample size and the corresponding limited statistical power, uncorrected *p*‐values < 0.05 are reported as *p*_*uncor*_, in bold with an asterisk (in Tables and Figures) or with a remark of whether they survive FDR‐correction in the text. Effect sizes were interpreted according to Funder and Ozer [49]: very small (*r* ≥ 0.05), small (*r* ≥ 0.10), medium (*r* ≥ 0.20), large (*r* ≥ 0.30), and very large (*r* ≥ 0.40). Partial effect sizes were interpreted according to Richardson [50]: small (*η* ^2^ = 0.01), medium (*η* ^2^ = 0.06), and large (*η* ^2^ = 0.14).

## 3 Results

The demographics of the final sample are presented in Table 2.

**TABLE 2:** Demographics of patients with PKU and controls.

		Patients, *n* = 30	Controls, *n* = 59	*U/χ* ^2^	*p*_*uncor*_	*r*_rb_/*φ*
**Age**	Median (IQR), years	35.5 (12.3)	30.3 (10.4)	790	0.412	−0.11
	Range, years	19–48	18–53			
**Sex**	Female, *n*	13	26	0.0	0.947	0.007
	Male, *n*	17	33			
**Education**	Median (IQR)	5.5 (3.8)	5 (3.5)	5.0	0.663	0.24

*Note:* Education was categorized into 1—Secondary Education; 2—Apprenticeship; 3—Vocational Education; 4—High School; 5—College of Higher Education; 6—Bachelor or equivalent; 7—Master or equivalent; 8—Doctorate. Uncorrected *p*‐values are indicated as *p*_*uncor*_. IQR = interquartile range; *U* = Mann–Whitney *U*‐statistic; *χ* ^2^ = Chi‐squared statistic; *r*_rb_ = rank‐biserial correlations as effect sizes for Mann–Whitney *U*‐tests; *φ* = phi as effect sizes for chi‐squared test.

**FIGURE 1:**
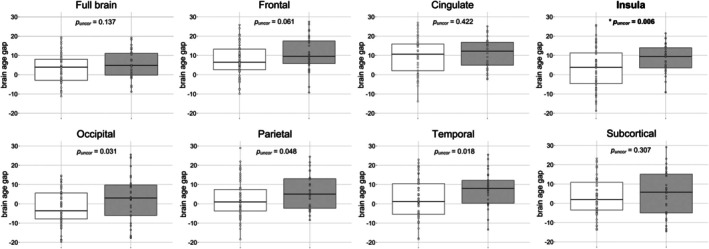
**Group differences between patients with PKU and controls in the brain age gap of the full brain and seven brain regions**. Patients with PKU are displayed in gray and controls in white. All *p*‐values are reported uncorrected (*p*_*uncor*_), with values surviving FDR‐correction highlighted in bold and with an asterisk.

Patients with PKU presented with a significantly higher BAG compared to healthy controls in the insula even after FDR‐correction, indicating older‐appearing brains in patients with PKU Figure 1; adjusted mean difference of 5.86 years (*p*_*uncor*_ = 0.006, 95% CI [1.71, 10.00], partial *η* ^2^ = 0.07; patients *M* = 8.70, 95% CI [5.32, 12.08]; controls *M* = 2.84, 95% CI [0.43, 5.25]). In three more brain regions, BAG was higher in patients than controls; however, these differences did not survive FDR‐correction (Figure 1).

The adjusted mean difference for the BAG in the occipital lobe was 4.80 years (95% CI [0.46, 9.14], *p*_*uncor*_ = 0.031, partial *η* ^2^ = 0.04) with patients (*M =* 2.79, 95% CI [−0.75, 6.33]) showing higher BAG in the occipital lobe and hence older‐appearing occipital brain regions compared to controls (*M =* −2.02, 95% CI [−4.54, 0.51]). The estimated adjusted mean difference for the BAG in the parietal lobe was 4.01 years (95% CI [0.04, 7.97], *p*_*uncor*_ = 0.048, partial *η* ^2^ = 0.04) with patients (*M =* 6.45, 95% CI [3.21, 9.68]) showing higher BAG in the parietal lobe compared to controls (*M =* 2.44, 95% CI [0.13, 4.75]). Lastly, the estimated adjusted mean difference for the BAG in the temporal lobe was 5.13 years (95% CI = [0.89, 9.36], *p*_*uncor*_ = 0.018, partial *η* ^2^ = 0.06) with patients (*M =* 7.72, 95% CI [4.260, 11.17]) showing higher BAG in the temporal lobe compared to controls (*M =* 2.59, 95% CI [0.12, 5.06]). No effects of group were found in the full brain, frontal lobe, cingulate, and subcortical regions.

Partial Spearman's correlations indicated some positive associations between the BAG in the cingulate cortex and concurrent blood Phe levels (*r*_*s*_ = 0.41, *p*_*uncor*_ = 0.029, 95% CI [0.03, 0.68]), historical Phe levels in adulthood (*r*_*s*_ = 0.68, *p*_*uncor*_ = 0.004, 95% CI [0.25, 0.90]), and lifetime Phe levels (*r*_*s*_ = 0.69, *p*_*uncor*_ = 0.013, 95% CI [0.13, 0.96]). These associations did not survive FDR‐correction (Figure S1). No further correlations between the BAGs and concurrent and historical Phe levels in patients were found (Table S2a and Table S2b), and no correlations between BAGs and Tyr and Trp occurred (Table S2a).

WM lesions were found in 29 out of 30 patients. The total WM lesion score ranged from 0 to 7 (media*n* = 2.5, IQR = 1.75). The most frequently affected areas were the parietal (*n* = 29) and occipital lobes (*n* = 19), followed by the frontal lobe (*n* = 15), temporal lobe (*n* = 4), brainstem (*n* = 3), and cerebellum (*n* = 2). One patient showed no WM lesions. In the remaining 29 patients, the total WM lesion scores ranged from 1 to 7, with the following distribution: 1 (6 patients), 2 (8 patients), 3 (7 patients), 4 (5 patients), 5 (2 patients), and 7 (1 patient). Descriptive statistics on the WM lesion rating have been reported elsewhere [51]. A positive association was found between the BAG in the cingulate and the total WM lesion score, but this correlation did not survive FDR correction. No further correlations between the BAG and the total WM lesion score occurred (Table 3).

**TABLE 3:** Correlations between BAG across all brain regions and total WM lesion score in patients with PKU.

Brain region	*r*_*s*_	95% CI	*p*_*uncor*_
Full brain	−0.08	[−0.40, 0.28]	0.675
Cingulate	0.40	[0.08, 0.68]	0.034
Frontal	0.00	[−0.40, 0.38]	0.985
Insula	0.06	[−0.39, 0.47]	0.767
Occipital	0.22	[−0.18, 0.60]	0.264
Parietal	0.06	[−0.32, 0.45]	0.743
Temporal	−0.25	[−0.58, 0.16]	0.209
Subcortical	−0.05	[−0.41, 0.33]	0.793

*Note:* None of the correlations survived FDR‐correction. Uncorrected *p*‐values are indicated as *p*_*uncor*_. *r*_*s*_ = Spearman's rank‐order correlation coefficient, CI = confidence interval.

Partial Spearman's correlations revealed some positive associations between the subcortical BAG and performance in inhibition (*r*_*s*_ = 0.38, *p*_*uncor*_ = 0.045, 95% CI [−0.01, 0.66]), and verbal fluency (*r*_*s*_ = −0.38, *p*_*uncor*_ = 0.047, 95% CI [−0.66, −0.04]). Additionally, cingulate BAG was negatively associated with performance in divided attention (*r*_*s*_ = −0.41, *p*_*uncor*_ = 0.034, 95% CI [−0.64, −0.04]). These associations did not survive FDR‐corrections (Figure S2). No further correlations between the BAGs and cognition in patients occurred (Table S3a).

Similarly, in controls, some associations occurred between verbal fluency and the subcortical BAG (*r*_*s*_ = −0.28, *p*_*uncor*_ = 0.039, 95% CI [−0.51, 0.02]), design fluency and the BAG in the occipital cortex (*r*_*s*_ = −0.36, *p*_*uncor*_ = 0.006, 95% CI [−0.58, −0.10]), and manual dexterity and the BAG in the cingulate cortex (*r*_*s*_ = 0.34, *p*_*uncor*_ = 0.009, 95% CI [0.10, 0.56]). Again, these associations did not survive FDR‐correction (Table S3b).

In patients with PKU, no correlations were found between BAG in different brain regions and mood (Table S4a).

In controls, some associations occurred between subcortical BAG and anxiety (*r*_*s*_ = 0.34, *p*_*uncor*_ = 0.010, 95% CI [0.08, 0.57]), fatigue (*r*_*s*_ = 0.29, *p*_*uncor*_ = 0.030, 95% CI [0.03, 0.52]), and total mood disturbance (*r*_*s*_ = 0.33, *p*_*uncor*_ = 0.012, 95% CI [0.07, 0.56]). These associations did not survive FDR corrections (Table S4b).

## 4 Discussion

In line with our hypothesis, adult patients with early‐treated PKU exhibited a higher BAG in the insula compared to healthy controls. The regional group differences found in the occipital, parietal, and temporal cortices did not survive FDR‐correction. No group differences were detected in full brain, frontal, cingulate, or subcortical BAG. Similarly, none of the associations between BAG and Phe levels in the PKU group, nor the associations with cognition, remained significant after correction for multiple comparisons.

Even though not all the regional effects remained statistically significant, the overall pattern suggests a region‐specific rather than global vulnerability to the long‐term consequences of PKU. The elevated BAG in the insula may reflect the cumulative impact of metabolic disturbances, such as prolonged or fluctuating exposure to elevated Phe levels, as well as the influence of life‐long dietary restrictions on brain structure. This interpretation aligns with previous findings showing that early dietary management may influence brain structure [2, 52].

Moreover, the insula's particular sensitivity to elevated Phe might be linked to its integral role in cognitive, sensory, and affective functions, including taste perception, working memory, and multisensory integration [53, 54]. Given that individuals with PKU adhere to a strict low‐protein diet, often leading to reduced gustatory stimulation [55], altered sensory processing may further contribute to accelerated, region‐specific structural alterations. These findings highlight the regional vulnerability of the insula as a potential neural marker of chronic metabolic and sensory burden in PKU.

Correlations between cingulate BAG and current or historical Phe levels did not survive FDR‐correction and therefore should be interpreted with caution. Nevertheless, the observed effect sizes could suggest a potential link between elevated Phe exposure and the structural integrity of the cingulate. Specifically, the effect‐sizes point towards a moderate to strong association between the cingulate BAG and current Phe levels (*r*_*s*_ = 0.41), and historical Phe levels in adulthood (*r*_*s*_ = 0.68) and over the lifetime (*r*_*s*_ = 0.69). These results are consistent with our previous work, where associations between brain structure and Phe levels did not survive FDR‐correction, neither in cross‐sectional analyses [4, 51] nor in an interventional trial investigating the effects of a 4‐week Phe elevation [29]. The lack of associations remaining statistically significant after FDR‐correction may be due to the small sample size or indicate that Phe alone does not exert a direct, linear effect on brain structure. Instead, the observed structural alterations and potentially elevated brain age may reflect the cumulative burden of broader metabolic disturbances associated with chronically elevated Phe levels. These could include imbalances in other amino acids, oxidative stress, neurotransmitter deficiencies, disruptions in myelination, or altered gut microbiota [56, 57]. It is worth noting, however, that other studies have found some positive associations between structural brain alterations and elevated Phe levels [58]. Further, brain alterations could also be due to changes in other metabolites and metabolic pathways (e.g., glutamate or purine metabolism), as shown in previous research with PKU mice. Such metabolite changes could entail energy dysregulation and oxidative stress [59, 60], a hypothesis that could ideally be further studied in a longitudinal setting.

In addition to gray matter alterations, abnormalities in the cerebral WM are frequently observed in PKU. In our sample, WM lesions were present in 29 out of 30 patients with PKU, most commonly in the parietal lobe, followed by the occipital and frontal lobes. According to previous research, WM hyperintensities in patients with PKU are assumed to reflect intramyelinic edema, a type of cytotoxic edema [61, 62]. Elevated Phe levels impact the permeability of membranes and may therefore directly underlie WM alterations [29, 63, 64]. Our results indicated a positive association between the BAG in the cingulate and WM lesion load (not surviving FDR‐correction) and align with previous findings showing a positive association between BAG and WM lesions in a healthy mid‐aged population [65]. Although this association should be interpreted with caution, our finding may nevertheless suggest a potential role of the cingulate cortex as an integrative region involving both frontal and parietal areas [66], where WM lesions in patients with PKU were most prevalent.

Finally, our results revealed weak associations between regional BAGs and cognitive performance in patients with PKU, none of which survived FDR‐correction. This aligns with previous studies reporting no [67] or only weak associations [68] between the BAG and cognitive performance in healthy adults. Therefore, patients with PKU might present with compensatory mechanisms that can obscure the relationship between structural features, such as cerebral brain age and cognitive performance. According to the biological aging perspective, cognitive decline emerges once compensatory mechanisms are exhausted [69, 70]. Brain maintenance refers to the preservation of brain integrity with aging or disease and is considered key to maintaining cognitive function [71]. This cognitive reserve capacity may help sustain cognitive function despite structural brain alterations as found in patients with PKU.

### 4.1 Limitations and Future Perspectives

Certain limitations should be acknowledged. First, the calculation of the BAG is subject to a systematic age bias due to the orthogonal proportion between estimated age and BAG (estimated age—chronological age). This leads to an overestimation of the brain age in younger individuals and an underestimation in older individuals [72, 73]. Bias correction models have yet to eliminate this issue [73]. In the present study, all analyses were corrected for age. However, the potential influence of age bias remains a concern, particularly since the study sample consisted primarily of younger and middle‐aged participants. This is also due to difficulties in recruiting individuals with a rare condition such as PKU. Further, the first early‐treated patients with PKU are now only entering their sixth decade of life, which makes it impossible to investigate brain age in early‐treated patients. Second, our results show a large inter‐individual variability in the BAG among both patients with PKU and controls. This could be explained by bio‐psycho‐social factors (e.g., lifestyle factors, birth weight, life satisfaction) and biomarkers not included in the present study, which may influence the BAG [21, 74, 75]. Although the BAG captures meaningful variance in brain structure, its interpretability is not absolute and the majority of the variance observed remains unexplained and therefore needs to be addressed in future longitudinal studies [75]. Third, the results reflect cross‐sectional findings, making it impossible to determine how the BAG changes over time in patients with PKU and whether potential differences from controls might increase or remain stable [76]. Further, we are not able to infer how Phe‐lowering therapies like Pegvaliase, Sapropterin, or Sepiapterin might impact brain aging. Again, longitudinal studies will enable better observations of brain age trajectories and, through this, improve predictions of clinical outcomes, especially for populations at risk for altered aging trajectories, such as patients with PKU. Fourth, given that the field of understanding brain aging is still rather new, issues of interpretability and translational validity remain relevant. This raises the key question: do patients with PKU present an older‐appearing insular brain region, or is the insula structurally vulnerable to PKU and elevated Phe levels and might therefore be interpreted as indicative of advanced brain age by the BAG metric.

### 4.2 Conclusion

This study provides valuable first insights into the potential influences of PKU on brain age. The observed elevation in insular BAG in adults with early‐treated PKU may reflect disease‐specific cerebral deviations resulting from the accumulated effects of early‐life or lifelong metabolic disturbances. BAG appears to capture PKU‐related alterations in specific brain regions and may therefore serve as a sensitive marker of disease‐related brain changes. The high reliability of the BAG observed in our sample further supports its potential utility for detecting deviations from healthy brain aging and improving prognostic assessments. Future longitudinal studies are needed to clarify the mechanisms underlying brain aging in PKU and the role of compensatory processes in preserving cognitive function despite structural alterations.

## Author Contributions

Conception and design: Regula Everts, Roman Trepp, and Raphaela Muri. Funding acquisition: Regula Everts, Roman Trepp, and Raphaela Muri. Data collection: Raphaela Muri. Data analysis: Raphaela Muri, Tobias Kaufmann, and Laura Winiger. Drafting of the article: Laura Winiger. Reviewing and editing the article: Regula Everts, Roman Trepp, Raphaela Muri, Tobias Kaufmann, Michel Hochuli, Emma Vardy, Piotr Radojewski, Katarzyna Pospieszny. Supervision: Regula Everts and Roman Trepp.

## Funding

The study was funded by the Swiss National Science Foundation (192706; 10002244), the Vontobel Foundation (Switzerland), the Bangerter Rhyner Foundation (Switzerland), the Fondation Rolf Gaillard pour la recherche en endocrinologie, diabétologie et métabolisme (Switzerland), the Maiores Foundation (Lichtenstein), and a Novartis Young Investigator Grant (Switzerland) awarded to Raphaela Muri. The funders had no involvement in the study design, data collection, analysis, or interpretation of the data.

## Ethics Statement

All authors were compliant and followed the ethical guidelines as required by the JIMD.

## Consent

All procedures followed were in accordance with the ethical standards of the responsible committee on human experimentation (institutional and national) and with the Helsinki Declaration of 1975, as revised in 2000. Informed consent was obtained from all participants before they were included in the study.

## Conflicts of Interest

Emma Vardy declares being a trustee of NSPKU and has received honoraria through Vitaflo. Laura Winiger, Raphaela Muri, Tobias Kaufmann, Michel Hochuli, Piotr Radojewski, Katarzyna Pospieszny, Roman Trepp, and Regula Everts declare no conflicts of interest.

## Supporting information


**Table S1a:** Correlations between the BAG across all brain regions and chronological age in patients with PKU and healthy controls.
**Table S1b:** Sex differences in BAGs across all brain regions in patients with PKU and controls.
**Table S2a:** Correlations between the BAG across all brain regions and concurrent metabolic parameters.
**Table S2b:** Correlations between the BAG across all brain regions and historical Phe values.
**Table S3a:** Correlations between BAG across all brain regions and cognitive performance in patients with PKU.
**Table S3b:** Correlations between BAG across all brain regions and cognitive performance in controls.
**Table S4a:** Correlations between BAG across all brain regions and mood in patients with PKU.
**Table S4b:** Correlations between BAG across all brain regions and mood in controls.
**Figure S1:** Correlations between BAG and concurrent and historical Phe.
**Figure S2:** Correlations between BAG and cognitive performance.

## Data Availability

The data that support the findings of this study are available on request from the corresponding author. The data are not publicly available due to privacy or ethical restrictions.
