# Supplementary material for: Brain Age in Adult Patients With Early‐Treated Phenylketonuria
Source: J Inherit Metab Dis. 2026 Feb 17;49(2):e70158. doi: 10.1002/jimd.70158 (PMC12913233; doi:10.1002/jimd.70158)
Supplement: Supplementary file 1 — Table S1a: Correlations between the BAG across all brain regions and chronological age in patients with PKU and healthy controls. Table S1b: Sex differences in BAGs across all brain regions in patients with PKU and controls. Table S2a: Correlations between the BAG across all brain regions and concurrent metabolic parameters. Table S2b: Correlations between the BAG across all brain regions and historical Phe values. Table S3a: Correlations between BAG across all brain regions and cognitive performance in patients with PKU. Table S3b: Correlations between BAG across all brain regions and cognitive performance in controls. Table S4a: Correlations between BAG across all brain regions and mood in patients with PKU. Table S4b: Correlations between BAG across all brain regions and mood in controls. Figure S1: Correlations between BAG and concurrent and historical Phe. Figure S2: Correlations between BAG and cognitive performance. [file JIMD-49-0-s001.docx]

**Supplementary material**

**Table S1a.** Correlations between the BAG across all brain regions and chronological age in patients with PKU and healthy controls

|  | **Patients** | | |  | **Controls** | | |
| --- | --- | --- | --- | --- | --- | --- | --- |
| **Brain region** | ***r_s_*** | **95% CI** | ***P_uncor_*** |  | ***r_s_*** | **95% CI** | ***P_uncor_*** |
| Fullbrain | –0.19 | [–0.51, 0.16] | 0.329 |  | –0.20 | [–0.42, 0.03] | 0.129 |
| Cingulate | –0.39 | [–0.70, –0.01] | 0.036 |  | –0.28 | [–0.53, 0.01] | 0.035 |
| Frontal | –0.31 | [–0.69, 0.12] | 0.104 |  | –0.07 | [–0.28, 0.15] | 0.607 |
| Insula | –0.24 | [–0.63, 0.20] | 0.200 |  | **–0.34** | **[–0.53, –0.09]** | **0.009*** |
| Occipital | –0.46 | [–0.72, –0.11] | 0.012 |  | **–0.32** | **[–0.52, –0.09]** | **0.014*** |
| Parietal | –0.25 | [–0.56, 0.10] | 0.188 |  | –0.12 | [–0.37, 0.14] | 0.353 |
| Temporal | –0.00 | [–0.35, 0.35] | 0.992 |  | –0.18 | [–0.40, 0.09] | 0.187 |
| Subcortical | –0.44 | [–0.71, –0.06] | 0.018 |  | **–0.46** | **[–0.64, –0.22]** | **<.001*** |
| *Note.* Correlations were performed for patients with PKU (*n* = 30), and healthy controls (*n* = 59). All *p*-values are reported uncorrected (*p_uncor_*), with values surviving FDR-correction highlighted in bold and with an asterisk. *r_s_ =* Spearman’s rank-order correlation coefficient, CI = Confidence interval. | | | | | | | |

**Table S1b.** Sex differences in BAGs across all brain regions in patients with PKU and controls

|  | **Patients** | | | |  | **Controls** | | | | |  |
| --- | --- | --- | --- | --- | --- | --- | --- | --- | --- | --- | --- |
| **Brain region** | **Estimate *(b)*** | **SE** | ***t*-value** | ***P_uncor_*** |  | **Estimate *(b)*** | **SE** | ***t*-value** | ***P_uncor_*** | | |
| Fullbrain | 0.91 | 3.70 | 0.25 | 0.808 |  | **5.15** | **1.99** | **2.58** | **0.012*** | | |
| Cingulate | 2.54 | 3.16 | 0.80 | 0.429 |  | 2.93 | 2.35 | 1.25 | 0.218 | | |
| Frontal | 0.25 | 3.40 | 0.07 | 0.942 |  | 3.37 | 2.36 | 1.43 | 0.159 | | |
| Insula | 2.46 | 3.24 | 0.76 | 0.453 |  | 7.13 | 2.89 | 2.47 | 0.017* | | |
| Occipital | 0.23 | 4.42 | 0.05 | 0.959 |  | –0.36 | 2.70 | –0.13 | 0.894 | | |
| Parietal | 2.96 | 3.77 | 0.78 | 0.439 |  | **6.55** | **2.36** | **2.78** | **0.007*** | | |
| Temporal | 0.49 | 3.65 | 0.13 | 0.894 |  | 4.72 | 2.75 | 1.72 | 0.092 | | |
| Subcortical | 2.62 | 4.91 | 0.53 | 0.598 |  | 4.16 | 2.43 | 1.71 | 0.093 | | |
| *Note.* Values are shown for *n* = 29 patients with PKU (female *n* = 13, male *n* = 16) and for *n* = 58 healthy controls (female n = 26, male n = 32). Reported values reflect the effect of sex (female). All *p*-values are reported uncorrected (*p_uncor_*), with values surviving FDR-correction highlighted in bold and with an asterisk Estimate (*b*) = Robust regression coefficients in years, SE = standard error. | | | | | | | | | |  |  |

**Table S2a.** Correlations between the BAG across all brain regions and concurrent metabolic parameters

| **Metabolic Parameter** | **Brain region** | ***r_s_*** | **95% CI** | ***P_uncor_*** | |
| --- | --- | --- | --- | --- | --- |
| **Phe** | Full brain | –0.01 | [–0.42, 0.38] | 0.973 | |
|  | Cingulate | 0.41 | [0.03, 0.68] | 0.029 | |
|  | Frontal | 0.13 | [–0.20, 0.42] | 0.519 | |
|  | Insula | –0.17 | [–0.53, 0.18] | 0.382 | |
|  | Occipital | –0.13 | [–0.49, 0.19] | 0.501 | |
|  | Parietal | –0.12 | [–0.57, 0.34] | 0.527 | |
|  | Temporal | 0.01 | [–0.47, 0.45] | 0.940 | |
|  | Subcortical | –0.15 | [–0.56, 0.21] | 0.456 | |
| **Tyr** | Full brain | 0.11 | [–0.27, 0.48] | 0.563 | |
|  | Cingulate | 0.29 | [–0.17, 0.63] | 0.135 | |
|  | Frontal | 0.22 | [–0.20, 0.60] | 0.252 | |
|  | Insula | 0.29 | [–0.09, 0.62] | 0.138 | |
|  | Occipital | 0.24 | [–0.18, 0.59] | 0.224 | |
|  | Parietal | 0.02 | [–0.35, 0.39] | 0.918 | |
|  | Temporal | 0.18 | [–0.21, 0.55] | 0.346 | |
|  | Subcortical | 0.12 | [–0.31, 0.52] | 0.528 | |
| **Trp** | Full brain | –0.23 | [–0.53, 0.14] | 0.233 | |
|  | Cingulate | 0.08 | [–0.35, 0.46] | 0.681 | |
|  | Frontal | –0.15 | [–0.53, 0.24] | 0.448 | |
|  | Insula | 0.06 | [–0.36, 0.42] | 0.749 | |
|  | Occipital | –0.20 | [–0.55, 0.22] | 0.318 | |
|  | Parietal | –0.14 | [–0.49, 0.22] | 0.483 | |
|  | Temporal | –0.33 | [–0.62, 0.04] | 0.089 | |
|  | Subcortical | –0.16 | [–0.50, 0.27] | 0.422 | |
| *Note.* None of the correlations survived FDR-correction. Uncorrected *p*-values are indicated as *p_uncor_.* Phe = Phenylalanin, Tyr = Tyrosin, Trp = Tryptophan. | | | | |  |

**Table S2b.** Correlations between the BAG across all brain regions and historical Phe values

| **Age categories Phe values** | **Brain region** | ***r_s_*** | **95% CI** | ***P_uncor_*** | |
| --- | --- | --- | --- | --- | --- |
| **IDC childhood 0-5** | Full brain | –0.14 | [–0.68, 0.55] | 0.631 | |
| *n* = 17 | Cingulate | 0.35 | [–0.24, 0.85] | 0.196 | |
|  | Frontal | –0.22 | [–0.84, 0.45] | 0.437 | |
|  | Insula | –0.34 | [–0.84, 0.29] | 0.218 | |
|  | Occipital | –0.27 | [–0.78, 0.28] | 0.324 | |
|  | Parietal | 0.14 | [–0.53, 0.71] | 0.630 | |
|  | Temporal | 0.00 | [–0.63, 0.65] | 0.989 | |
|  | Subcortical | 0.04 | [–0.54, 0.56] | 0.891 | |
| **IDC childhood 6-12** | Full brain | –0.35 | [–0.72, 0.17] | 0.172 | |
| *n* = 19 | Cingulate | 0.26 | [–0.32, 0.74] | 0.315 | |
|  | Frontal | –0.38 | [–0.84, 0.14] | 0.131 | |
|  | Insula | –0.43 | [–0.83, 0.04] | 0.087 | |
|  | Occipital | –0.19 | [–0.66, 0.34] | 0.469 | |
|  | Parietal | –0.12 | [–0.58, 0.39] | 0.660 | |
|  | Temporal | –0.23 | [–0.72, 0.29] | 0.370 | |
|  | Subcortical | –0.09 | [–0.55, 0.45] | 0.745 | |
| **IDC adolescence 13-17** | Full brain | –0.22 | [–0.64, 0.30] | 0.356 | |
| *n* = 21 | Cingulate | 0.17 | [–0.30, 0.66] | 0.490 | |
|  | Frontal | –0.40 | [–0.78, 0.07] | 0.088 | |
|  | Insula | –0.42 | [–0.73, 0.03] | 0.077 | |
|  | Occipital | –0.45 | [–0.76, 0.05] | 0.051 | |
|  | Parietal | –0.15 | [–0.64, 0.44] | 0.528 | |
|  | Temporal | –0.31 | [–0.76, 0.24] | 0.201 | |
|  | Subcortical | 0.31 | [–0.21, 0.71] | 0.192 | |
| **IDC adulthood ≥ 18** | Full brain | –0.22 | [–0.77, 0.37] | 0.405 | |
| *n* = 18 | Cingulate | 0.68 | [0.25, 0.90] | 0.004 | |
|  | Frontal | –0.18 | [–0.82, 0.40] | 0.494 | |
|  | Insula | –0.22 | [–0.78, 0.31] | 0.423 | |
|  | Occipital | –0.13 | [–0.70, 0.41] | 0.636 | |
|  | Parietal | 0.12 | [–0.61, 0.63] | 0.657 | |
|  | Temporal | –0.14 | [–0.78, 0.41] | 0.604 | |
|  | Subcortical | –0.12 | [–0.62, 0.38] | 0.661 | |
| **IDC lifetime** | Full brain | –0.25 | [–0.88, 0.50] | 0.436 | |
| *n* = 14 | Cingulate | 0.69 | [0.13, 0.96] | 0.013 | |
|  | Frontal | –0.31 | [–0.88, 0.27] | 0.319 | |
|  | Insula | –0.31 | [–0.85, 0.28] | 0.324 | |
|  | Occipital | –0.49 | [–0.88, 0.11] | 0.102 | |
|  | Parietal | 0.14 | [–0.68, 0.76] | 0.656 | |
|  | Temporal | –0.10 | [–0.88, 0.66] | 0.755 | |
|  | Subcortical | –0.01 | [–0.68, 0.65] | 0.983 | |
| *Note.* None of the correlations survived FDR-correction. Uncorrected *p*-values are indicated as *p_uncor_.* Historical Phenylalanine (Phe) values are shown in five different age categories. IDC = Index of dietary control. | | | | |  |

**Table S3a.** Correlations between BAG across all brain regions and cognitive performance in patients with PKU

| **Cognitive variable** | **Brain region** | ***r_s_*** | **95% CI** | ***P_uncor_*** | |
| --- | --- | --- | --- | --- | --- |
| **General intelligence** | Full brain | –0.29 | [–0.63, 0.09] | 0.128 | |
|  | Cingulate | 0.11 | [–0.28, 0.51] | 0.566 | |
|  | Frontal | –0.21 | [–0.54, 0.16] | 0.279 | |
|  | Insula | –0.26 | [–0.61, 0.14] | 0.177 | |
|  | Occipital | 0.21 | [–0.18, 0.53] | 0.283 | |
|  | Parietal | –0.09 | [–0.45, 0.26] | 0.657 | |
|  | Temporal | –0.23 | [–0.54, 0.13] | 0.244 | |
|  | Subcortical | –0.19 | [–0.61, 0.22] | 0.339 | |
| **Working memory**^a^ | Full brain | 0.02 | [–0.38, 0.39] | 0.921 | |
|  | Cingulate | 0.02 | [–0.37, 0.44] | 0.912 | |
|  | Frontal | 0.13 | [–0.30, 0.46] | 0.495 | |
|  | Insula | 0.15 | [–0.32, 0.53] | 0.449 | |
|  | Occipital | 0.26 | [–0.09, 0.57] | 0.180 | |
|  | Parietal | 0.15 | [–0.29, 0.58] | 0.434 | |
|  | Temporal | –0.23 | [–0.61, 0.17] | 0.230 | |
|  | Subcortical | 0.02 | [–0.35, 0.39] | 0.930 | |
| **Inhibition** | Full brain | 0.37 | [–0.05, 0.72] | 0.056 | |
|  | Cingulate | –0.01 | [–0.44, 0.40] | 0.963 | |
|  | Frontal | 0.05 | [–0.41, 0.50] | 0.815 | |
|  | Insula | –0.14 | [–0.51, 0.31] | 0.482 | |
|  | Occipital | 0.05 | [–0.34, 0.46] | 0.816 | |
|  | Parietal | 0.00 | [–0.41, 0.43] | 0.994 | |
|  | Temporal | 0.13 | [–0.25, 0.51] | 0.525 | |
|  | Subcortical | 0.38 | [–0.01, 0.66] | 0.045 | |
| **Cognitive flexibility** | Full brain | 0.21 | [–0.25, 0.66] | 0.278 | |
|  | Cingulate | –0.08 | [–0.54, 0.36] | 0.679 | |
|  | Frontal | 0.12 | [–0.36, 0.58] | 0.550 | |
|  | Insula | 0.14 | [–0.35, 0.61] | 0.479 | |
|  | Occipital | –0.05 | [–0.45, 0.34] | 0.781 | |
|  | Parietal | –0.08 | [–0.51, 0.38] | 0.675 | |
|  | Temporal | –0.02 | [–0.42, 0.42] | 0.926 | |
|  | Subcortical | 0.28 | [–0.11, 0.61] | 0.148 | |
| **Alertness** | Full brain | 0.14 | [–0.27, 0.48] | 0.475 | |
|  | Cingulate | –0.16 | [–0.46, 0.17] | 0.420 | |
|  | Frontal | –0.16 | [–0.52, 0.30] | 0.415 | |
|  | Insula | –0.24 | [–0.59, 0.23] | 0.213 | |
|  | Occipital | –0.11 | [–0.53, 0.35] | 0.578 | |
|  | Parietal | –0.22 | [–0.54, 0.21] | 0.261 | |
|  | Temporal | 0.00 | [–0.40, 0.40] | 0.998 | |
|  | Subcortical | 0.03 | [–0.37, 0.38] | 0.887 | |
| **Divided attention** | Full brain | 0.05 | [–0.35, 0.43] | 0.804 | |
|  | Cingulate | –0.41 | [–0.64, -0.04] | 0.034 | |
|  | Frontal | –0.15 | [–0.52, 0.27] | 0.448 | |
|  | Insula | 0.02 | [–0.38, 0.46] | 0.910 | |
|  | Occipital | 0.16 | [–0.29, 0.54] | 0.435 | |
|  | Parietal | –0.10 | [–0.47, 0.30] | 0.606 | |
|  | Temporal | –0.03 | [–0.40, 0.38] | 0.882 | |
|  | Subcortical | 0.24 | [–0.15, 0.57] | 0.229 | |
| **Sustained attention** | Full brain | –0.01 | [–0.45, 0.43] | 0.966 | |
|  | Cingulate | –0.28 | [–0.60, 0.13] | 0.159 | |
|  | Frontal | –0.18 | [–0.52, 0.21] | 0.370 | |
|  | Insula | –0.15 | [–0.52, 0.30] | 0.449 | |
|  | Occipital | –0.04 | [–0.45, 0.37] | 0.831 | |
|  | Parietal | 0.00 | [–0.39, 0.38] | 0.993 | |
|  | Temporal | 0.12 | [–0.31,0.53] | 0.545 | |
|  | Subcortical | 0.06 | [–0.36, 0.51] | 0.757 | |
| **Verbal fluency tot** | Full brain | –0.18 | [–0.56, 0.19] | 0.346 | |
|  | Cingulate | –0.05 | [–0.46, 0.36] | 0.802 | |
|  | Frontal | 0.10 | [–0.31, 0.46] | 0.627 | |
|  | Insula | –0.02 | [–0.45, 0.36] | 0.910 | |
|  | Occipital | 0.02 | [–0.35, 0.40] | 0.909 | |
|  | Parietal | –0.08 | [–0.47, 0.34] | 0.701 | |
|  | Temporal | –0.12 | [–0.47, 0.27] | 0.550 | |
|  | Subcortical | –0.38 | [–0.66, -0.04] | 0.047 | |
| **Manual dexterity** | Full brain | –0.24 | [–0.54, 0.14] | 0.224 | |
|  | Cingulate | 0.27 | [–0.14, 0.64] | 0.168 | |
|  | Frontal | –0.11 | [–0.51, 0.28] | 0.581 | |
|  | Insula | –0.04 | [–0.44, 0.36] | 0.830 | |
|  | Occipital | 0.05 | [–0.29, 0.41] | 0.785 | |
|  | Parietal | –0.07 | [–0.48, 0.32] | 0.722 | |
|  | Temporal | –0.19 | [–0.50, 0.17] | 0.338 | |
|  | Subcortical | –0.22 | [–0.51, 0.11] | 0.260 | |
| **Working memory**^b^ | Full brain | –0.14 | [–0.50, 0.23] | 0.480 | |
|  | Cingulate | 0.14 | [–0.23, 0.52] | 0.484 | |
|  | Frontal | –0.06 | [–0.49, 0.34] | 0.751 | |
|  | Insula | 0.07 | [–0.35, 0.51] | 0.728 | |
|  | Occipital | –0.09 | [–0.46, 0.33] | 0.658 | |
|  | Parietal | 0.12 | [–0.27, 0.45] | 0.550 | |
|  | Temporal | 0.01 | [–0.39, 0.41] | 0.947 | |
|  | Subcortical | –0.19 | [–0.51, 0.16] | 0.335 | |
| **Design fluency** | Full brain | 0.15 | [–0.25, 0.49] | 0.447 | |
|  | Cingulate | 0.26 | [–0.17, 0.58] | 0.173 | |
|  | Frontal | 0.21 | [–0.18, 0.50] | 0.284 | |
|  | Insula | –0.07 | [–0.50, 0.35] | 0.730 | |
|  | Occipital | 0.27 | [–0.13, 0.59] | 0.170 | |
|  | Parietal | 0.10 | [–0.34, 0.49] | 0.625 | |
|  | Temporal | –0.10 | [–0.48, 0.30] | 0.617 | |
|  | Subcortical | 0.02 | [–0.37, 0.41] | 0.933 | |
| *Note*. None of the correlations survived FDR-correction. Uncorrected *p*-values are indicated as *p_uncor_.* ^a^ Working memory measured with the n-back task of the Test of Attentional Performance (TAP). ^b^ Working memory measured with the subtest letter-number sequencing of the Wechsler Adult Intelligence Scale Fourth Edition (WAIS-IV). | | | | |  |

**Table S3b.** Correlations between BAG across all brain regions and cognitive performance in controls

| **Cognitive variable** | **Brain region** | | ***r_s_*** | | **95% CI** | | ***P_uncor_*** | |  |
| --- | --- | --- | --- | --- | --- | --- | --- | --- | --- |
| **General intelligence** | Full brain | | –0.18 | | [–0.45, 0.11] | | 0.177 | |  |
|  | Cingulate | | –0.02 | | [–0.31, 0.27] | | 0.898 | |  |
|  | Frontal | | –0.16 | | [–0.41, 0.10] | | 0.250 | |  |
|  | Insula | | –0.08 | | [–0.33, 0.20] | | 0.542 | |  |
|  | Occipital | | –0.13 | | [–0.41, 0.15] | | 0.330 | |  |
|  | Parietal | | –0.20 | | [–0.43, 0.07] | | 0.144 | |  |
|  | Temporal | | –0.22 | | [–0.49, 0.07] | | 0.105 | |  |
|  | Subcortical | | –0.22 | | [–0.48, 0.07] | | 0.106 | |  |
| **Working memory**^a^ | Full brain | | 0.05 | | [–0.23, 0.32] | | 0.720 | |  |
|  | Cingulate | | 0.03 | | [–0.26, 0.30] | | 0.847 | |  |
|  | Frontal | | –0.04 | | [–0.32, 0.25] | | 0.794 | |  |
|  | Insula | | 0.15 | | [–0.12, 0.40] | | 0.283 | |  |
|  | Occipital | | –0.04 | | [–0.32, 0.25] | | 0.789 | |  |
|  | Parietal | | 0.10 | | [–0.15, 0.36] | | 0.464 | |  |
|  | Temporal | | 0.00 | | [–0.30, 0.30] | | 0.994 | |  |
|  | Subcortical | | –0.11 | | [–0.34, 0.15] | | 0.424 | |  |
| **Inhibition** | Full brain | | –0.07 | | [–0.36, 0.22] | | 0.595 | |  |
|  | Cingulate | | –0.25 | | [–0.49, 0.04] | | 0.062 | |  |
|  | Frontal | | –0.04 | | [–0.29, 0.22] | | 0.779 | |  |
|  | Insula | | –0.14 | | [–0.40, 0.13] | | 0.318 | |  |
|  | Occipital | | 0.11 | | [–0.16, 0.37] | | 0.414 | |  |
|  | Parietal | | 0.00 | | [–0.29, 0.29] | | 0.988 | |  |
|  | Temporal | | 0.04 | | [–0.24, 0.29] | | 0.782 | |  |
|  | Subcortical | | 0.01 | | [–0.27, 0.27] | | 0.914 | |  |
| **Cognitive flexibility** | Full brain | | –0.07 | | [–0.35, 0.22] | | 0.626 | |  |
|  | Cingulate | | –0.07 | | [–0.32, 0.20] | | 0.620 | |  |
|  | Frontal | | –0.08 | | [–0.34, 0.20] | | 0.564 | |  |
|  | Insula | | –0.03 | | [–0.29, 0.22] | | 0.809 | |  |
|  | Occipital | | 0.03 | | [–0.26, 0.30] | | 0.820 | |  |
|  | Parietal | | 0.04 | | [–0.24, 0.30] | | 0.783 | |  |
|  | Temporal | | –0.05 | | [–0.31, 0.24] | | 0.731 | |  |
|  | Subcortical | | 0.03 | | [–0.24, 0.29] | | 0.834 | |  |
| **Alertness** | Full brain | | 0.16 | | [–0.09, 0.38] | | 0.241 | |  |
|  | Cingulate | | –0.08 | | [–0.35, 0.18] | | 0.560 | |  |
|  | Frontal | | 0.13 | | [–0.14, 0.37] | | 0.356 | |  |
|  | Insula | | 0.09 | | [–0.16, 0.33] | | 0.497 | |  |
|  | Occipital | | 0.13 | | [–0.14, 0.38] | | 0.356 | |  |
|  | Parietal | | 0.01 | | [–0.24, 0.25] | | 0.944 | |  |
|  | Temporal | | 0.10 | | [–0.17, 0.37] | | 0.449 | |  |
|  | Subcortical | | 0.22 | | [–0.06, 0.47] | | 0.105 | |  |
| **Divided attention** | Full brain | | 0.13 | | [–0.18, 0.40] | | 0.348 | |  |
|  | Cingulate | | 0.00 | | [–0.27, 0.27] | | 0.987 | |  |
|  | Frontal | | 0.08 | | [–0.19, 0.36] | | 0.538 | |  |
|  | Insula | | 0.06 | | [–0.21, 0.33] | | 0.654 | |  |
|  | |  | |  | |  | |  | |
|  | | Occipital | | 0.02 | | [–0.29, 0.31] | | 0.900 | |
|  | Parietal | | 0.13 | | [–0.16, 0.40] | | 0.356 | |  |
|  | Temporal | | 0.12 | | [–0.17, 0.38] | | 0.367 | |  |
|  | Subcortical | | 0.12 | | [–0.17, 0.39] | | 0.380 | |  |
| **Sustained attention** | Full brain | | 0.13 | | [–0.15, 0.39] | | 0.336 | |  |
|  | Cingulate | | –0.15 | | [–0.42, 0.14] | | 0.281 | |  |
|  | Frontal | | 0.08 | | [–0.22, 0.36] | | 0.555 | |  |
|  | Insula | | 0.04 | | [–0.21, 0.29] | | 0.754 | |  |
|  | Occipital | | 0.24 | | [–0.02, 0.46] | | 0.080 | |  |
|  | Parietal | | 0.02 | | [–0.22, 0.25] | | 0.859 | |  |
|  | Temporal | | 0.14 | | [–0.11, 0.40] | | 0.297 | |  |
|  | Subcortical | | 0.18 | | [–0.09, 0.44] | | 0.174 | |  |
| **Verbal fluency tot** | Full brain | | –0.11 | | [–0.38, 0.18] | | 0.407 | |  |
|  | Cingulate | | 0.04 | | [–0.25, 0.32] | | 0.789 | |  |
|  | Frontal | | –0.05 | | [–0.31, 0.22] | | 0.690 | |  |
|  | Insula | | 0.10 | | [–0.16, 0.35] | | 0.472 | |  |
|  | Occipital | | –0.04 | | [–0.32, 0.22] | | 0.777 | |  |
|  | Parietal | | –0.06 | | [–0.33, 0.22] | | 0.664 | |  |
|  | Temporal | | –0.08 | | [–0.37, 0.23] | | 0.541 | |  |
|  | Subcortical | | –0.28 | | [–0.51, 0.02] | | 0.039 | |  |
| **Manual dexterity** | Full brain | | 0.18 | | [–0.09, 0.44] | | 0.180 | |  |
|  | Cingulate | | 0.34 | | [0.10, 0.56] | | 0.009 | |  |
|  | Frontal | | 0.22 | | [–0.06, 0.47] | | 0.107 | |  |
|  | Insula | | 0.22 | | [–0.07, 0.48] | | 0.104 | |  |
|  | Occipital | | 0.15 | | [–0.16, 0.44] | | 0.255 | |  |
|  | Parietal | | 0.12 | | [–0.16, 0.40] | | 0.364 | |  |
|  | Temporal | | 0.15 | | [–0.10, 0.40] | | 0.272 | |  |
|  | Subcortical | | –0.04 | | [–0.30, 0.24] | | 0.763 | |  |
| **Working memory**^b^ | Full brain | | –0.02 | | [–0.30, 0.27] | | 0.869 | |  |
|  | Cingulate | | 0.03 | | [–0.24, 0.31] | | 0.825 | |  |
|  | Frontal | | 0.02 | | [–0.25, 0.28] | | 0.895 | |  |
|  | Insula | | 0.10 | | [–0.18, 0.36] | | 0.473 | |  |
|  | Occipital | | 0.15 | | [–0.14, 0.43] | | 0.282 | |  |
|  | Parietal | | –0.08 | | [–0.35, 0.21] | | 0.578 | |  |
|  | Temporal | | –0.15 | | [–0.43, 0.15] | | 0.257 | |  |
|  | Subcortical | | –0.05 | | [–0.31, 0.23] | | 0.723 | |  |
| **Design fluency** | Full brain | | –0.18 | | [–0.45, 0.11] | | 0.183 | |  |
|  | Cingulate | | 0.07 | | [–0.21, 0.35] | | 0.608 | |  |
|  | Frontal | | –0.20 | | [–0.44, 0.05] | | 0.146 | |  |
|  | Insula | | –0.12 | | [–0.36, 0.17] | | 0.373 | |  |
|  | Occipital | | –0.36 | | [–.58, –0.10] | | 0.006 | |  |
|  | Parietal | | –0.22 | | [–0.47, 0.05] | | 0.101 | |  |
|  | Temporal | | –0.19 | | [–0.46, 0.10] | | 0.157 | |  |
|  | Subcortical | | –0.17 | | [–0.39, 0.06] | | 0.202 | |  |
| *Note.* None of the correlations survived FDR-correction. Uncorrected *p*-values are indicated as *p_uncor_.* ^a^ Working memory measured with the n-back task of the Test of Attentional Performance (TAP). ^b^ Working memory measured with the subtest letter-number sequencing of the Wechsler Adult Intelligence Scale Fourth Edition (WAIS-IV). | | | | | | | | | |

**Table S4a.** Correlations between BAG across all brain regions and mood in patients with PKU

| **Mood Scale** | **Brain region** | | ***r_s_*** | | **95% CI** | | ***P_uncor_*** | |
| --- | --- | --- | --- | --- | --- | --- | --- | --- |
| **Depression^a^** | Full brain | 0.11 | | [–0.28, 0.48] | | 0.583 | |  |
|  | Cingulate | 0.18 | | [–0.29, 0.56] | | 0.360 | |  |
|  | Frontal | 0.28 | | [–0.15, 0.63] | | 0.152 | |  |
|  | Insula | 0.15 | | [–0.33, 0.53] | | 0.453 | |  |
|  | Occipital | –0.18 | | [–0.53, 0.22] | | 0.372 | |  |
|  | Parietal | 0.06 | | [–0.29, 0.40] | | 0.768 | |  |
|  | Temporal | 0.24 | | [–0.11, 0.55] | | 0.215 | |  |
|  | Subcortical | –0.14 | | [–0.52, 0.30] | | 0.479 | |  |
| **Anxiety^b^** | Full brain | –0.14 | | [–0.58, 0.32] | | 0.488 | |  |
|  | Cingulate | 0.18 | | [–0.17, 0.49] | | 0.349 | |  |
|  | Frontal | 0.14 | | [–0.25, 0.54] | | 0.466 | |  |
|  | Insula | –0.14 | | [–0.52, 0.27] | | 0.478 | |  |
|  | Occipital | –0.31 | | [–0.62, 0.10] | | 0.109 | |  |
|  | Parietal | –0.11 | | [–0.47, 0.30] | | 0.593 | |  |
|  | Temporal | –0.03 | | [-0.42, 0.37] | | 0.873 | |  |
|  | Subcortical | –0.33 | | [–0.71, 0.12] | | 0.087 | |  |
| **Vigor**^b^ | Full brain | –0.05 | | [–0.40, 0.33] | | 0.810 | |  |
|  | Cingulate | 0.27 | | [–0.08, 0.61] | | 0.167 | |  |
|  | Frontal | 0.09 | | [–0.31, 0.44] | | 0.658 | |  |
|  | Insula | 0.16 | | [–0.28, 0.52] | | 0.422 | |  |
|  | Occipital | –0.12 | | [–0.46, 0.26] | | 0.557 | |  |
|  | Parietal | 0.18 | | [–0.20, 0.52] | | 0.353 | |  |
|  | Temporal | –0.26 | | [–0.57, 0.11] | | 0.184 | |  |
|  | Subcortical | –0.04 | | [–0.43, 0.36] | | 0.842 | |  |
| **Fatigue**^b^ | Full brain | 0.13 | | [–0.29, 0.53] | | 0.520 | |  |
|  | Cingulate | 0.32 | | [–0.01, 0.58] | | 0.098 | |  |
|  | Frontal | 0.17 | | [–0.16, 0.52] | | 0.375 | |  |
|  | Insula | 0.01 | | [–0.38, 0.43] | | 0.974 | |  |
|  | Occipital | –0.19 | | [–0.52, 0.20] | | 0.323 | |  |
|  | Parietal | –0.01 | | [–0.40, 0.40] | | 0.952 | |  |
|  | Temporal | 0.28 | | [–0.08, 0.60] | | 0.142 | |  |
|  | Subcortical | –0.10 | | [–0.52, 0.35] | | 0.614 | |  |
| **Anger**^b^ | Full brain | 0.07 | | [–0.35, 0.45] | | 0.732 | |  |
|  | Cingulate | 0.27 | | [–0.10, 0.54] | | 0.162 | |  |
|  | Frontal | 0.20 | | [–0.21, 0.56] | | 0.299 | |  |
|  | Insula | –0.07 | | [–0.44, 0.31] | | 0.725 | |  |
|  | Occipital | 0.01 | | [–0.40, 0.46] | | 0.951 | |  |
|  | Parietal | 0.09 | | [–0.27, 0.45] | | 0.651 | |  |
|  | Temporal | 0.05 | | [–0.32, 0.41] | | 0.804 | |  |
|  | Subcortical | –0.14 | | [–0.58, 0.25] | | 0.466 | |  |
| **TMD**^c^ | Full brain | 0.05 | | [–0.34, 0.43] | | 0.797 | |  |
|  | Cingulate | 0.04 | | [–0.34, 0.33] | | 0.856 | |  |
|  | Frontal | 0.09 | | [–0.28, 0.47] | | 0.634 | |  |
|  | Insula | –0.06 | | [–0.44, 0.36] | | 0.753 | |  |
|  | Occipital | 0.00 | | [–0.38, 0.36] | | 0.986 | |  |
|  | Parietal | –0.07 | | [–0.44, 0.32] | | 0.710 | |  |
|  | Temporal | 0.25 | | [–0.10, 0.57] | | 0.193 | |  |
|  | Subcortical | –0.12 | | [–0.53, 0.29] | | 0.534 | |  |
| *Note*. None of the correlations survived FDR-correction. Uncorrected *p*-values are indicated as *p_uncor_.* ^a^ Beck Depression Inventory (BDI-II) total score. ^b^ Score of Profile of Mood States (POMS; anxiety, vigor, fatigue, and hostility).^c^ TMD = Total mood disturbance. | | | | | | | |  |

**Table S4b.** Correlations between BAG across all brain regions and mood in controls

| **Mood Scale** | | **Brain region** | ***r_s_*** | **95% CI** | ***P_uncor_*** |
| --- | --- | --- | --- | --- | --- |
| **Depression**^a^ | Full brain | | –0.15 | [–0.40, 0.12] | 0.278 |
|  | Cingulate | | 0.00 | [–0.26, 0.28] | 0.994 |
|  | Frontal | | –0.23 | [–0.46, 0.03] | 0.090 |
|  | Insula | | –0.21 | [–0.42, 0.03] | 0.116 |
|  | Occipital | | –0.12 | [–0.39, 0.15] | 0.359 |
|  | Parietal | | –0.08 | [–0.33, 0.17] | 0.535 |
|  | Temporal | | –0.06 | [–0.30, 0.19] | 0.639 |
|  | Subcortical | | –0.03 | [–0.29, 0.24] | 0.841 |
| **Anxiety**^b^ | Full brain | | 0.16 | [–0.11, 0.40] | 0.242 |
|  | Cingulate | | 0.05 | [–0.23, 0.35] | 0.704 |
|  | Frontal | | 0.00 | [–0.27, 0.28] | 0.979 |
|  | Insula | | –0.04 | [–0.30, 0.22] | 0.749 |
|  | Occipital | | 0.09 | [–0.20, 0.34] | 0.525 |
|  | Parietal | | 0.09 | [–0.17, 0.33] | 0.527 |
|  | Temporal | | 0.06 | [–0.22, 0.32] | 0.678 |
|  | Subcortical | | 0.34 | [0.08, 0.57] | 0.010 |
| **Vigor**^b^ | Full brain | | –0.09 | [–0.35, 0.18] | 0.508 |
|  | Cingulate | | –0.21 | [–0.48, 0.08] | 0.119 |
|  | Frontal | | –0.08 | [–0.34, 0.18] | 0.563 |
|  | Insula | | –0.12 | [–0.39, 0.16] | 0.390 |
|  | Occipital | | 0.06 | [–0.19, 0.32] | 0.652 |
|  | Parietal | | –0.14 | [–0.41, 0.14] | 0.296 |
|  | Temporal | | –0.24 | [–0.44, -0.01] | 0.072 |
|  | Subcortical | | –0.09 | [–0.35, 0.16] | 0.510 |
| **Fatigue**^b^ | Full brain | | 0.11 | [–0.16, 0.38] | 0.396 |
|  | Cingulate | | 0.01 | [–0.27, 0.32] | 0.941 |
|  | Frontal | | 0.05 | [–0.22, 0.31] | 0.733 |
|  | Insula | | 0.07 | [–0.21, 0.35] | 0.608 |
|  | Occipital | | 0.24 | [0.00, 0.46] | 0.069 |
|  | Parietal | | 0.04 | [–0.22, 0.29] | 0.772 |
|  | Temporal | | 0.12 | [–0.15, 0.37] | 0.392 |
|  | Subcortical | | 0.29 | [0.03, 0.52] | 0.030 |
| **Anger**^b^ | Full brain | | 0.19 | [–0.06, 0.43] | 0.148 |
|  | Cingulate | | 0.11 | [–0.16, 0.36] | 0.421 |
|  | Frontal | | 0.01 | [–0.26, 0.28] | 0.926 |
|  | Insula | | 0.18 | [–0.07, 0.40] | 0.170 |
|  | Occipital | | 0.24 | [–0.01, 0.49] | 0.067 |
|  | Parietal | | 0.14 | [–0.12, 0.37] | 0.315 |
|  | Temporal | | 0.19 | [–0.07, 0.42] | 0.152 |
|  | Subcortical | | 0.19 | [–0.08, 0.44] | 0.151 |
| **TMD**^c^ | Full brain | | 0.18 | [–0.09, 0.44] | 0.172 |
|  | Cingulate | | 0.10 | [–0.17, 0.39] | 0.457 |
|  | Frontal | | 0.02 | [–0.26, 0.30] | 0.872 |
|  | Insula | | 0.08 | [–0.18, 0.34] | 0.531 |
|  | Occipital | | 0.17 | [–0.10, 0.43] | 0.208 |
|  | Parietal | | 0.12 | [–0.16, 0.36] | 0.387 |
|  | Temporal | | 0.17 | [–0.09, 0.41] | 0.204 |
|  | Subcortical | | 0.33 | [0.07, 0.56] | 0.012 |
| *Note*. None of the correlations survived FDR-correction. Uncorrected *p*-values are indicated as *p_uncor_.* ^a^ Beck Depression Inventory (BDI-II) total score. ^b^ Score of Profile of Mood States (POMS; anxiety, vigor, fatigue, and hostility, TMD). | | | | | |


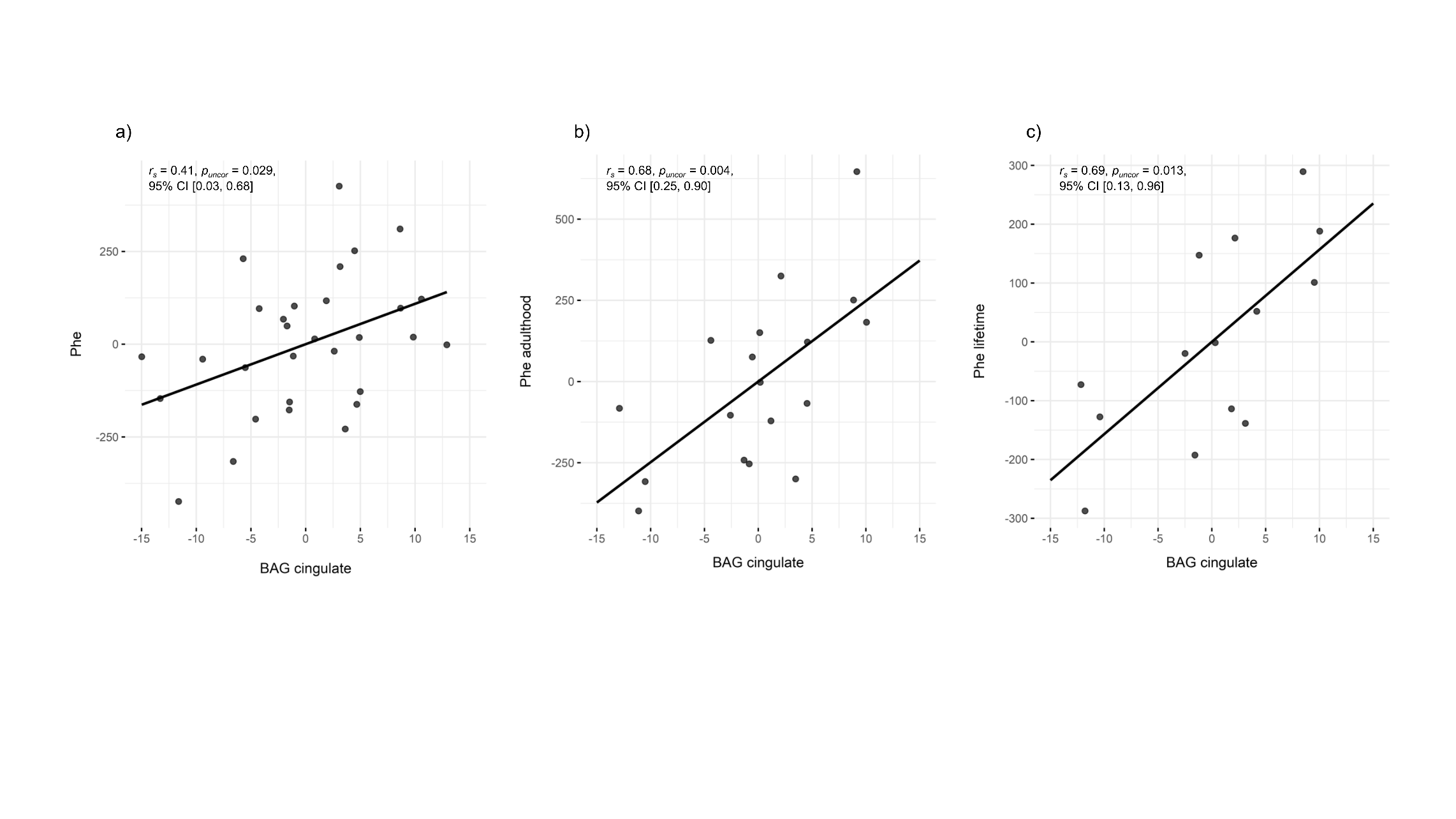


**Figure S1. Correlations between BAG and concurrent and historical Phe**

Values are displayed as residuals adjusted for age and sex. Residuals for Phe are shown as μmol/L, and residuals for BAG are shown in years. None of the correlations survived FDR correction. Uncorrected *p*-values are indicated as *p_uncor_.* **a)** Correlation between BAG and concurrent Phe levels, n = 30. **b)** Correlation between BAG and Phe levels in adulthood, n = 18. **c)** Correlation between BAG and lifetime Phe levels, n = 14.

Phe = Phenylalanine*, r_s_* = Spearman’s rank correlation coefficient*,* CI = confidence interval.

**
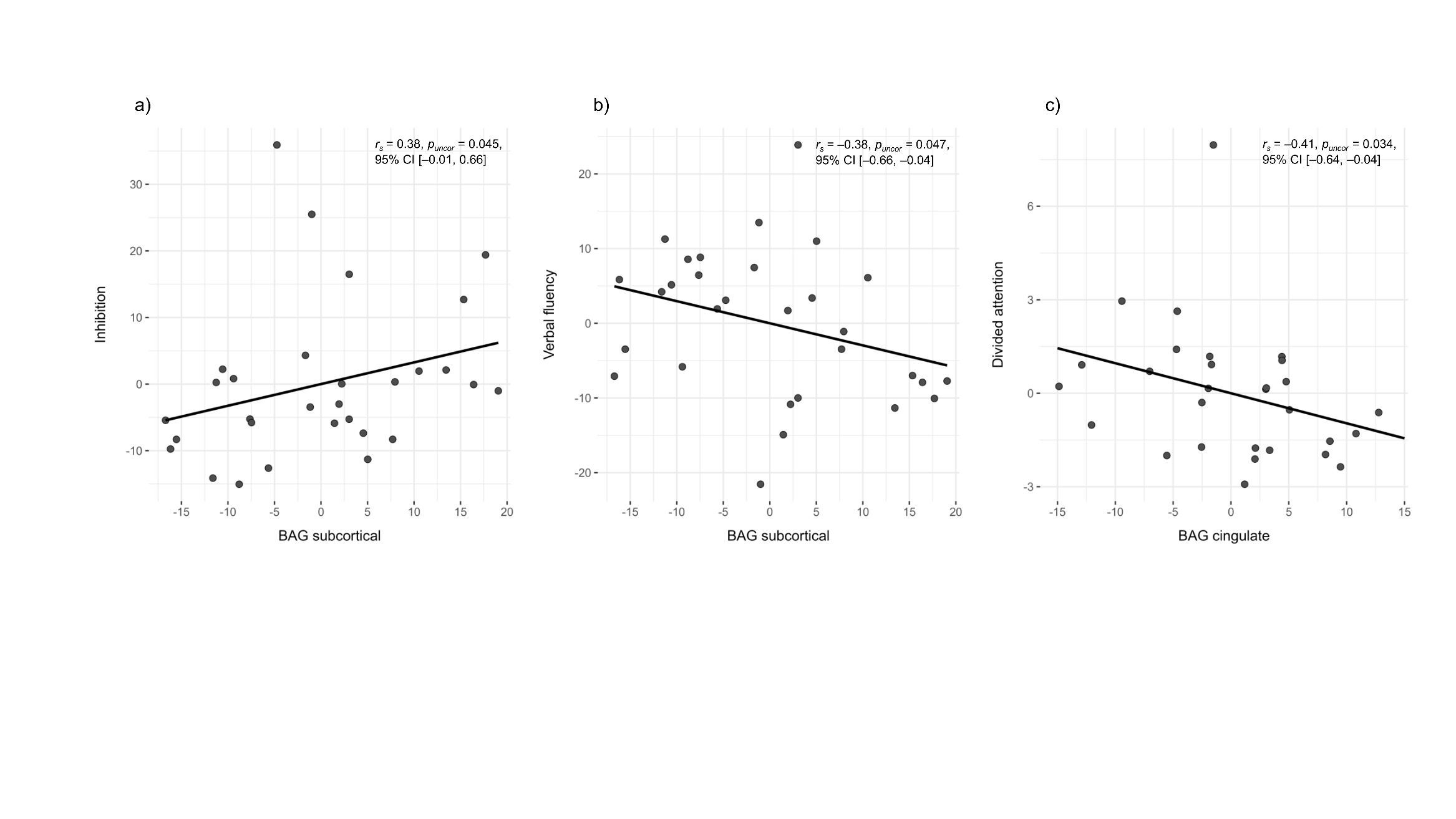
**

**Figure S2. Correlations between BAG and cognitive performance**

Values are displayed as residuals adjusted for age and sex. Residual scores for BAG are shown in years. None of the correlations survived FDR-correction. Uncorrected *p*-values are indicated as *p_uncor_.* **a)** Residual scores for inhibition are shown in seconds, n = 30. **b)** Residual scores for verbal fluency are shown in numbers of correct generated words, n = 30. **c)** Residual scores for divided attention are shown in total numbers of omissions, n = 29.

*r_s_* = Spearman’s rank correlation coefficient*,* CI = confidence interval.
